# Cryo-EM structure of gas vesicles for buoyancy-controlled motility

**DOI:** 10.1016/j.cell.2023.01.041

**Published:** 2023-03-02

**Authors:** Stefan T. Huber, Dion Terwiel, Wiel H. Evers, David Maresca, Arjen J. Jakobi

**Affiliations:** 1 Department of Bionanoscience, Kavli Institute of Nanoscience, Delft University of Technology, Delft 2628CD, the Netherlands; 2 Department of Imaging Physics, Delft University of Technology, Delft 2628CD, the Netherlands

**Keywords:** cryo-EM, gas vesicle, GvpA, GvpC, microbial motility, acoustic reporter gene

## Abstract

Gas vesicles are gas-filled nanocompartments that allow a diverse group of bacteria and archaea to control their buoyancy. The molecular basis of their properties and assembly remains unclear. Here, we report the 3.2 Å cryo-EM structure of the gas vesicle shell made from the structural protein GvpA that self-assembles into hollow helical cylinders closed off by cone-shaped tips. Two helical half shells connect through a characteristic arrangement of GvpA monomers, suggesting a mechanism of gas vesicle biogenesis. The fold of GvpA features a corrugated wall structure typical for force-bearing thin-walled cylinders. Small pores enable gas molecules to diffuse across the shell, while the exceptionally hydrophobic interior surface effectively repels water. Comparative structural analysis confirms the evolutionary conservation of gas vesicle assemblies and demonstrates molecular features of shell reinforcement by GvpC. Our findings will further research into gas vesicle biology and facilitate molecular engineering of gas vesicles for ultrasound imaging.

## Highlights

• 3.2 Å cryo-EM structure of microbial gas vesicles reveals atomic model of vesicle shell
• The structure explains the molecular basis of selective permeability and vesicle growth
• Fold and assembly are highly conserved between evolutionarily distant species
• Binding interface and geometry of secondary structural protein GvpC are identified

## Graphical abstract

**Figure:**
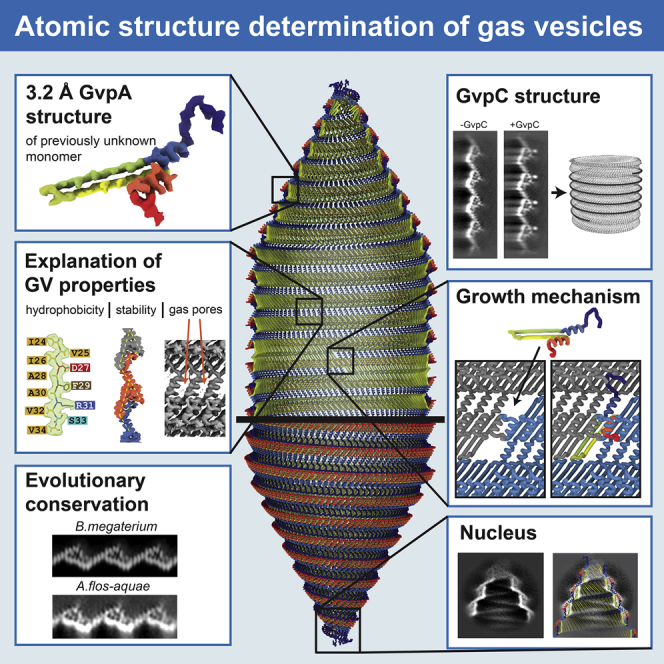

## Teaser

Cryo-EM structure of the gas vesicle shell provides insights into the evolutionarily conserved mechanistic basis of gas vesicle biogenesis and selective permeability.

## Introduction

Microorganisms utilize active motility systems to move toward or away from a variety of environmental stimuli such as chemicals and light.^1^ These include swimming by rotation of rigid flagella and movement over solid surfaces with filamentous appendages.^2^ Other forms of motility do not rely on active propulsion. Aquatic bacteria and archaea have evolved mechanisms to regulate buoyancy and can—similar to ballast tanks in submarines—create and eliminate gas-filled compartments to allow vertical migration in the water column. The cellular compartments providing positive buoyancy are formed by gas-filled protein shells called gas vesicles (GVs).^3^

There are very specific requirements for such structures: to achieve net buoyancy, GVs must occupy a substantial proportion of the cell, which involves forming compartments that extend over hundreds of nanometers in size. To maximize buoyancy the shell must be constructed from minimal material. At the same time, the shell needs to provide resistance to hydrostatic pressure to maintain buoyancy with changes in water depth.^4^

GVs have therefore evolved as rigid, thin-walled structures composed of a single protein unit that typically polymerizes into large cylindrical shells closed off by conical tips.^5^^,^^6^ The shell allows gas to diffuse passively between the GV lumen and the surrounding liquid, while effectively repelling water.^7^ All GVs identified to date appear to be constructed from the same components.^8^ The ∼7 kDa primary GV protein GvpA forms the core of the GV shell and the cone-shaped tips. A second protein, GvpC, binds the exterior of the GV and provides additional structural reinforcement.^9^^,^^10^

With molar masses exceeding hundreds of MDa, GVs range among the largest protein-based macromolecular assemblies reported to date. Despite intensive efforts,^5^^,^^11^^,^^12^^,^^13^^,^^14^^,^^15^^,^^16^^,^^17^ the molecular structure of GVs and therefore a molecular-level understanding of their distinctive properties have remained elusive. Here, we present the cryogenic electron microscopy (cryo-EM) structure of the canonical GV shell, providing detailed insight into the biogenesis of GVs and the unique evolutionary adaptions that enable buoyancy-controlled motility.

## Results

### Cryo-EM structure of the gas vesicle wall

**Figure 1:**
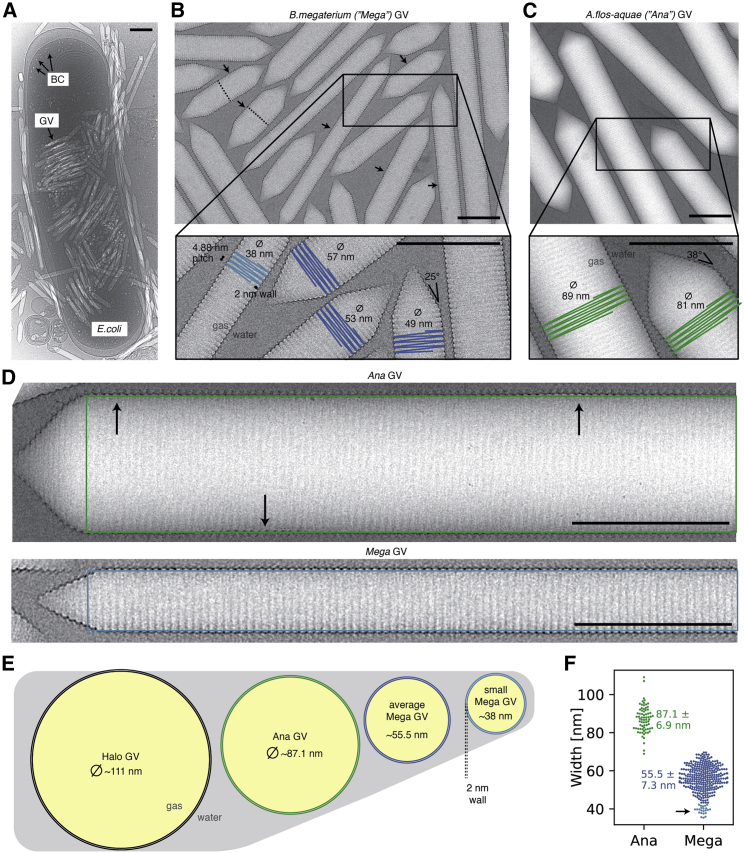
Gas vesicles (A) Cryo-EM micrograph of an *E. coli* cell heterologously producing *B. megaterium* gas vesicles (GVs). Mature GVs and small bicone (BC) nuclei are visible inside bacteria and in the surrounding medium. (B) Cryo-EM micrograph of purified *B. megaterium* GVs. GVs appear brighter than the surrounding solvent due to the lower density of the gas inside the GV lumen. Dashed lines on a subset of GVs represent midpoints of the helical GV segment; arrows indicate the location of the seam. The inset shows close-up examples of average-sized GVs (blue) and a small diameter GV (light blue) used for structure determination. The 4.88 Å helical pitch, the 2 nm-thick wall, and the 25° cone angle are annotated. (C) Representative cryo-EM micrograph of Ana GVs with inset showing a close-up indicating diameters and the ∼38° cone angle. (D) *A. flos-aquae* (Ana) GVs with mean diameter of 87 nm deform in cryo-EM sample preparation and do not stay perfectly cylindrical. The green rectangle depicts the ideal, non-deformed shape of an ideal cylinder in projection. Arrows point at deviations of the GV from the ideal shape. Helical reconstruction in cryo-EM assumes perfect helical crystals of the repeating unit. This assumption is violated when GVs “squish” during sample preparation. Very thin *B. megaterium* (Mega) GVs maintain a cylindrical shape in the thin ice layer of the cryo-EM sample. (E) Schematic cross-sections of *H. salinarum*, *A. flos-aquae*, and *B.megaterium* GVs drawn to scale. Average diameter of *H. salinarum* GV from Dutka et al.^18^ (F) Width measurements in 20 micrographs from both *A. flos-aquae* and *B. megaterium* GV datasets (this study). A long tail of small-diameter outliers with 34–42 nm diameter (black arrow) was observed in *B. megaterium* GVs. All scale bars 100 nm.

We expressed and purified *Bacillus megaterium* GVs that form narrow tubes most suitable for cryo-EM analysis (Figure 1). The small diameter enables preparation of samples with thin ice to maximize contrast and reduces the propensity of GVs to deform from ideal cylindrical shape. The native *B. megaterium* GV gene cluster contains two almost identical GvpA homologs, named GvpA and GvpB; for consistency in naming convention, we will refer to them as GvpA1 and GvpA2. A minimal gene cluster including GvpA2 but lacking GvpA1 is sufficient for GV assembly in *E. coli*^19^^,^^20^ (Figure 1A). Cryo-EM images showed GVs forming 0.1–1 μm long cylinders with varying diameters (55 ± 7 nm), consistent with previous data.^18^ A subset of images (∼16%) contained GVs with diameters as small as 34–42 nm (Figure 1B), which had their cylinder shape best preserved in the thin liquid film of cryo-EM samples (Figures 1B–1F).

**Figure S1:**
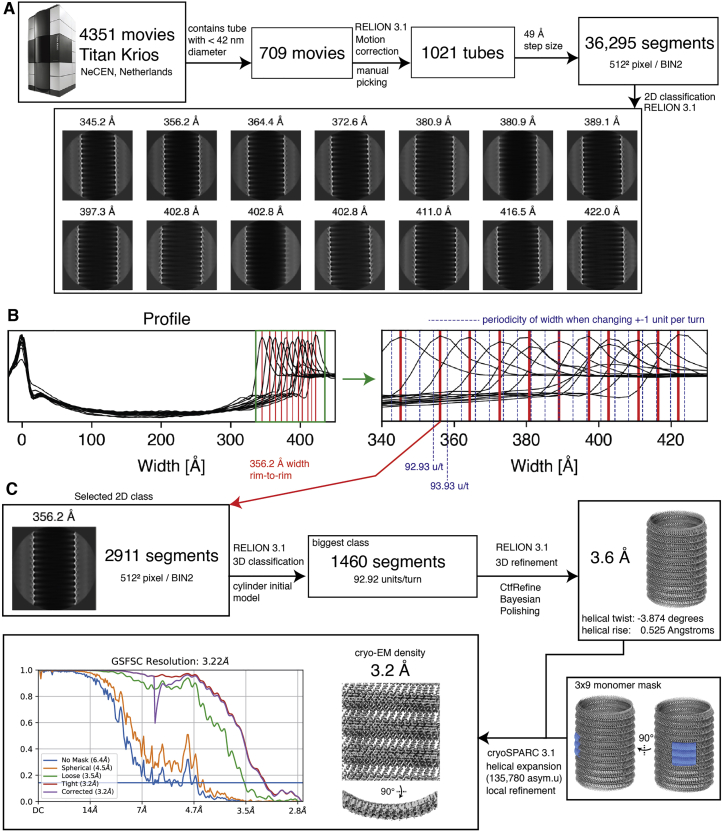
Data processing of *B. megaterium* gas vesicle dataset, related to Figure 2 (A) Preprocessing, manual picking, segment extraction and 2D classification leads to 2D class averages of GVs with different diameters. (B) The 2D classes were projected along the helical axis to generate profiles. The profiles were aligned with respect to the left peak. Zooming into the right peaks shows the distribution of GV widths in the 2D classes. Peaks are marked with a vertical red line. Blue lines indicate the periodicity of widths when an increment of one monomer per helical turn is assumed, based on a side-to-side distance of monomers of 12 Å, leading to a diameter increment of 12/π = 3.8 Å. Two to three different helical polymorphs are part of the particle subset belonging to a single 2D class average. (C) Processing steps starting from 2911 selected segments of a particular 2D class. The particle subset from the 2D class was further selected by 3D classification, imposing possible symmetry candidates between 90 and 95 units per wrung to select 1460 segments. Focused refinement on a 3x9 monomer segment of the wall in cryoSPARC 3.1^21^ after symmetry expansion to 135,780 asymmetric units leads to the final result of a 3.2 Å resolution cryo-EM density of the GV wall.

**Figure 2:**
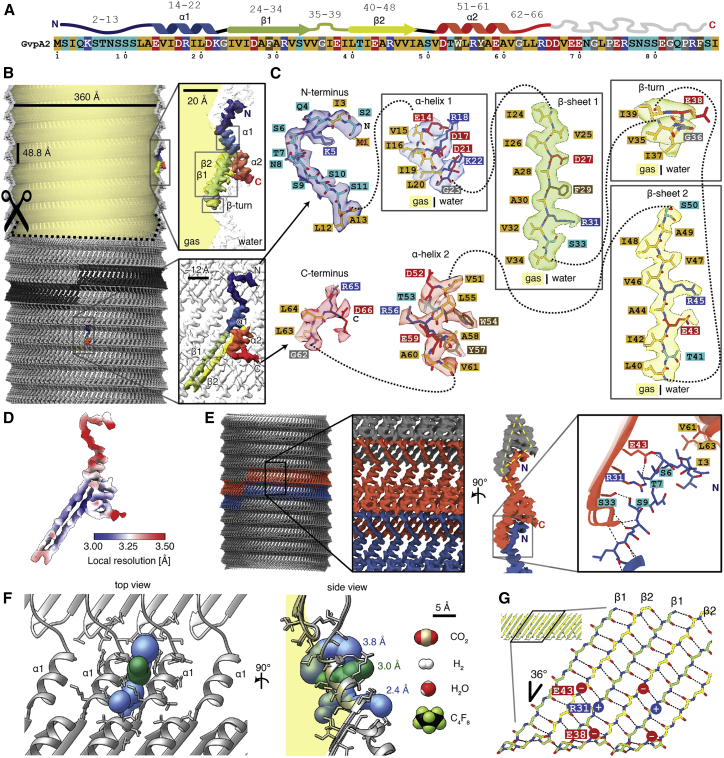
Cryo-EM structure of the gas vesicle wall (A) Primary and secondary structure of *B. megaterium* GvpA2. Residues in the primary structure are colored based on physicochemical properties. (B) GvpA2 monomers form thin-walled gas-filled protein shells assembled into a left-handed helix. One individual rib formed by 93 monomers is highlighted in dark gray. The top part of the GV is cut open to visualize the gas space. The 3.2 Å resolution cryo-EM density is shown with a single monomer colored according to sequence (N terminus, blue; C terminus, red) from a side view (top inset) and front view (bottom inset). (C) Entire cryo-EM density of a GvpA2 monomer annotated with respective amino acid one-letter-codes and the atomic model. Aliphatic residues (Ala, Val, Leu, Ile) line the gas-facing side of the GV wall. (D) Estimate of local resolution mapped onto the monomer density. (E) Cryo-EM density of the GV wall with two ribs highlighted in orange and blue. Side view illustrating corrugated zigzag structure and triangular cross-sections of the GV wall (yellow lines). Close up of inter-rib interactions mediated by the N terminus (blue), which binds across the β-hairpin and the C terminus (orange) of adjacent ribs, stabilized by hydrogen bonds from backbone, polar side chains (Ser_6_, Thr_7_, Ser_9_), and hydrophobic contacts (Ile_3_). (F) A slit between α1 helices allows diffusion of gas through the wall. Three computed tunnels approximate the slit and have bottleneck sizes ranging from 2.4 to 3.8 Å. The van der Waals surfaces of several gas molecules known to diffuse through the wall are shown to scale, with perfluorocyclobutane being the largest with 6.3 Å collision diameter.^7^ (G) Schematic of the β strand rib providing most lateral connections for the assembly through backbone hydrogen bonding (dotted lines) and electrostatic interactions (Glu_43_ -Arg_31_ -Glu_38_). See also Figure S1, Table S1, and Video S1.

We used a combination of two- and three-dimensional (2D and 3D) classification techniques to select 4% of the small GV subset corresponding to 35.6 nm diameter (Figure S1) and used helical reconstruction to obtain a cryo-EM density of 3.2 Å resolution (Figure 2). The final reconstruction yielded a cylindrical GV shell assembly with ∼93 GvpA2 monomers per helical turn, which represents one member of a range of helical polymorphs with diameters ranging from 34 to 70 nm and with 90–183 monomers per helical turn.

The reconstructed density allowed *de novo* atomic model building of GvpA2 in the structural context of its native assembly (Figure 2; Table S1). The cylindrical shell is constructed from thousands of GvpA2 monomers polymerized side by side into ribs spiraling into a left-handed helix with a helical pitch of 48.8 Å and −3.87° helical twist, resulting in 92.93 GvpA2 units per helical turn (Figures 2A and 2B; Video S1). Contrary to postulated models,^14^^,^^16^ GvpA monomers and not antiparallel dimers form the repeating units of the helical assembly. GvpA2 adopts a coil-α-β-β-α-coil fold (Figures 2A and 2B). The carboxyl-terminal residues Asp_67_-Ile_88_ are flexible and not resolved in our structure. The helical lattice forms an array of ribs consisting of densely packed GvpA2 subunits with a lateral center-to-center distance of ∼12 Å. The central β-hairpin, tilted at −36° relative to the long axis of the cylinder, forms the core of the GV ribs. Helix α2 folds back onto the hairpin, and helix α1 forms a bridge across the ∼16 Å gap separating adjacent ribs. The GV wall is therefore only one or two peptide layers thick (Figure 2B). The inner wall of the GV shell forms a continuous hydrophobic surface consisting of a dense pattern of hydrophobic residues located on the luminal side of the β-hairpin and helix α1 (Figures 2C and 2D). Connections between the ribs of the GV shell are formed by the predominantly polar N terminus, which extends perpendicular to helix α1 and folds across the β-hairpin of the adjacent rib, stabilized by interactions with several residues in the β-hairpin and helix α2 (Figure 2E).

Video S1. Overview of the cryo-EM reconstruction of *B. megaterium* GvpA, related to Figure 2 The movie highlights rib-to-rib contacts and luminal hydrophobicity and shows the monomer structure in the cryo-EM density.

The extreme hydrophobicity of the luminal GV surface constitutes an energetic barrier for diffusion of liquid water or condensation of gaseous H_2_O. Consistently, GVs have been shown to be impermeable to water but to be highly permeable to gas molecules.^7^ How gas molecules pass through the GV wall has so far been unclear. We located pores in the GV shell formed by slit-like openings between α1 helices of adjacent GpvA2 monomers (Figure 2F). We quantified the resulting pore size in the GV assembly computationally using Voronoi diagrams^22^ and retrieved three different access routes with minimal constrictions ranging from 2.4 to 3.8 Å, compatible with the collisional cross-sections of gases dissolved in the cytosol (Figure 2F).^23^

Despite its limited thickness, the GV shell can resist several atmospheres of pressure without collapse.^4^ GvpA2 monomers are held together tightly by lateral connections along the GV ribs formed by an extensive hydrogen-bonding network between the β strand backbones (Figure 2G). The hydrogen bonds are oriented at an angle of 54° relative to the cylinder axis, which is close to the "magic angle" 54.7° at which transverse and longitudinal stresses are equal in the wall of a cylinder.^3^^,^^24^ Additional reinforcements are made by a continuous network of salt bridges formed by Glu_43_-Arg_31_ between two monomers and Arg_31_-Glu_38_ within a monomer. The GvpA2 shell consists of alternating line segments and triangular cross-sections providing force-bearing elements (Figure 2E). The corrugations increase stiffness along the rib direction, while the linear segments provide compliant hinge elements that increases elasticity of GVs, increasing their capacity to accommodate deformations orthogonal to the rib without collapse.^25^

### Gas vesicles consist of two half shells with inverted orientation

**Figure 3:**
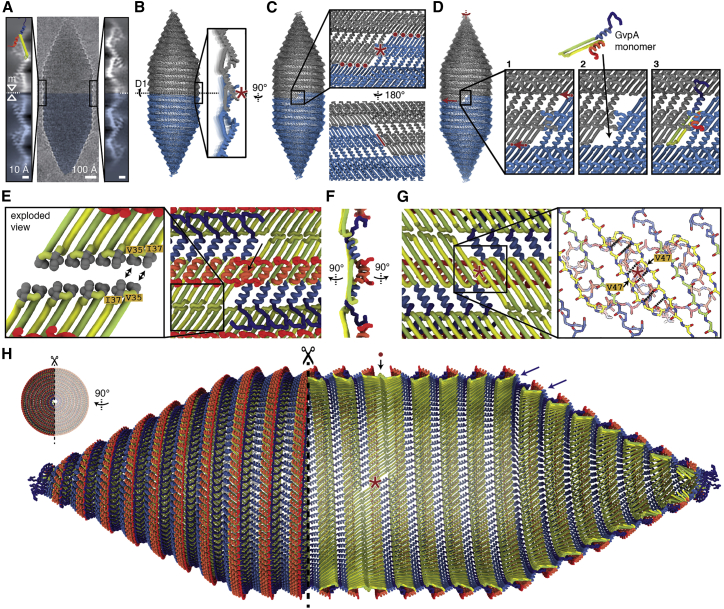
Pseudo-atomic model of a gas vesicle with two half shells in inverted orientation (A) Raw cryo-EM image of a single GV (with inverted contrast). β-hairpins of GvpA2 (cartoon) always point toward the seam at the center of the GV cylinder. Two different types of 2D class averages of the seam (left and right) are observed. The mirror symmetry (mirror axis: m) suggests a 180° symmetry axis (D1) at the point where two inversely oriented GV half shells meet. (B) Pseudo-atomic model of a GV constructed from two identical halves (gray, blue) with close up showing a side view of the polarity reversal point (PRP, red asterisk). (C) Close-up view onto the GvpA2 lattice around the PRP (red asterisk). Red dots indicate molecular contacts along the GV circumference where β-turns contact. The red line indicates contact between parts of hairpin strands β2 at the PRP. (D) Model of monomer insertion at the PRP. The two GV halves rotate against each other, with the β-hairpin contacts sliding over each other (red arrow) in a ratcheting fashion to allow monomer insertion in the resulting gap. Insertion of the monomer in the orientation opposite to that depicted is geometrically equivalent and would enlarge the other GV half shell. (E) View onto the seam between the two GV half shells with color scheme as in Figure 2B. The seam is sealed by contacting β-turns and the hydrophobic side chains Val35 and Ile37 (exploded view in inset). The lateral orientation of the hairpins is not uniquely determined by the data. An unresolved sterical clash between α2 helices from GvpA monomers around the PRP is marked by an arrow. (F) Side view of the PRP with color scheme as in Figure 2B. (G) View of the PRP from inside the GV. Oppositely oriented monomers next to the PRP are connected by six hydrogen bonds formed between the backbones of the β2 strands, with amino acid Val47 being located around the D1 symmetry axis (red asterisk). (H) Enlarged, sideways-rotated, and cut-open GV model with PRP (asterisk) and seam (dot) annotated. The proposed hinging motion of the N terminus required for adaption to the cylinder-to-cone transition and the reducing diameter of the tip is shown (blue arrows). See also Figures S2 and S3.

**Figure S2:**
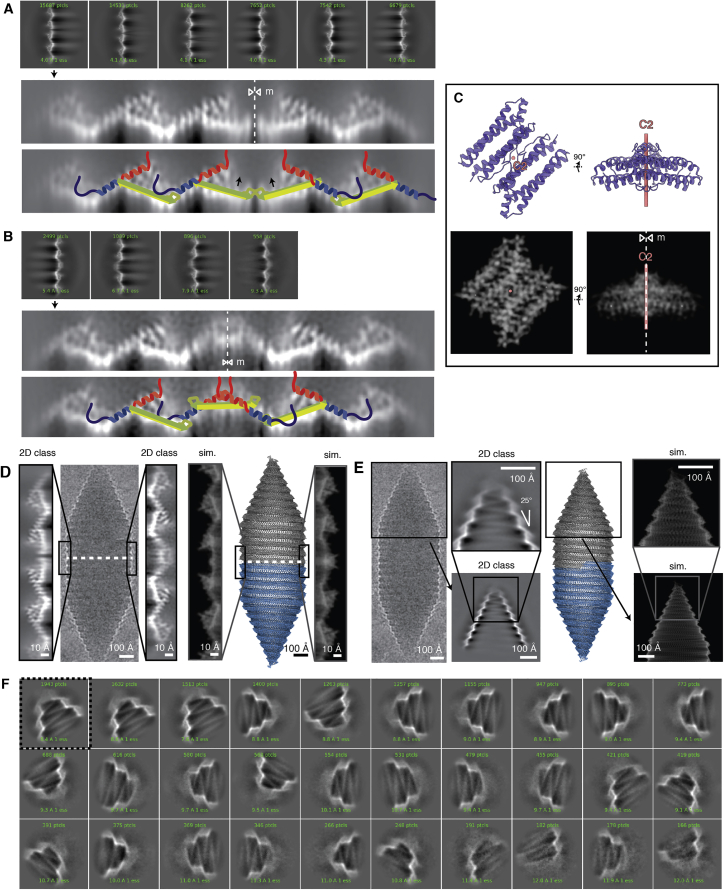
2D classification of *B. megaterium* seams and tips, related to Figure 3 (A) 2D classes from the seam show perfect or near-perfect mirror symmetry. β-hairpins seem to hinge upwards at the seam (black arrows). (B) 2D classes from the putative PRP. Selected classes were magnified and sharpened for easier depiction. The mirror line is shown (m, dotted white line). A cartoon is drawn on the 2D classes to visualize GvpA molecules with the N-terminus in blue and the C-terminus in red. (C) Demonstration of the fact that views orthogonal to a 180° rotation axis show mirror symmetry in projection. Apoferritin dimer (PDB: 7ohf) is shown along the C2 axis and orthogonal to the C2 axis. 2D projection images (right) orthogonal to the C2 axis show mirror symmetry. (D) 2D class from the seam and PRP and simulated EM density from the pseudo-atomic model are in close agreement. (E) Pseudo-atomic model of a GV with simulated 2D projections of the tip, closely matching the experimental data. The 2D class average of GV tips with large box size reveals a linear decrease in radius at the tips with a cone angle of 25°. (F) 2D classes of GV tips with smaller box size reveal more detail, but all end in a blurry density at the tip. Alignment of secondary structure features is not possible and indicated strong structural heterogeneity at the tips.

The thin film of the cryo-EM sample orients the large GVs into a sideways orientation, providing a consistent viewing direction onto the GV edges in projection. Detailed inspection of GV edges revealed that GvpA2 monomers are always oriented with their β-turns pointing toward the center of the GV cylinder (Figure 3A), which contains a structural irregularity that has previously been referred to as a seam.^13^ 2D class averages of GV edges around this seam showed two oppositely oriented GvpA2 monomers that make contact via their β-turns (Figures 3A and S2A) implying that this is the contact site of two GV half shells with inverted orientation.

While two contacting cylinders or cones have the same contact geometry along the entire circumference, GVs are assembled from two contacting helices. Continuity of the helicity implies there must be a unique polarity reversal point (PRP) at a point along the circumference, where an upwards- and a downwards-oriented GvpA2 monomer meet side by side. Apart from classes showing contacting β-turns, there is one less frequent set of 2D classes of the seam that displays two overlapping GvpA2 monomers in inverted orientation (Figures 3A and S2B). We posit that this 2D class is a projection view of the PRP located at the GV edge. As GVs can freely rotate around the cylinder axis in the thin ice layer, the PRP will be located exactly at the edge only at special rotation angles, explaining why this class is observed less frequently. The mirror symmetry in the 2D class implies a viewing direction perpendicular to a 180° (C2/D1) rotation axis (Figure S2C) that points through the PRP.

**Figure S3:**
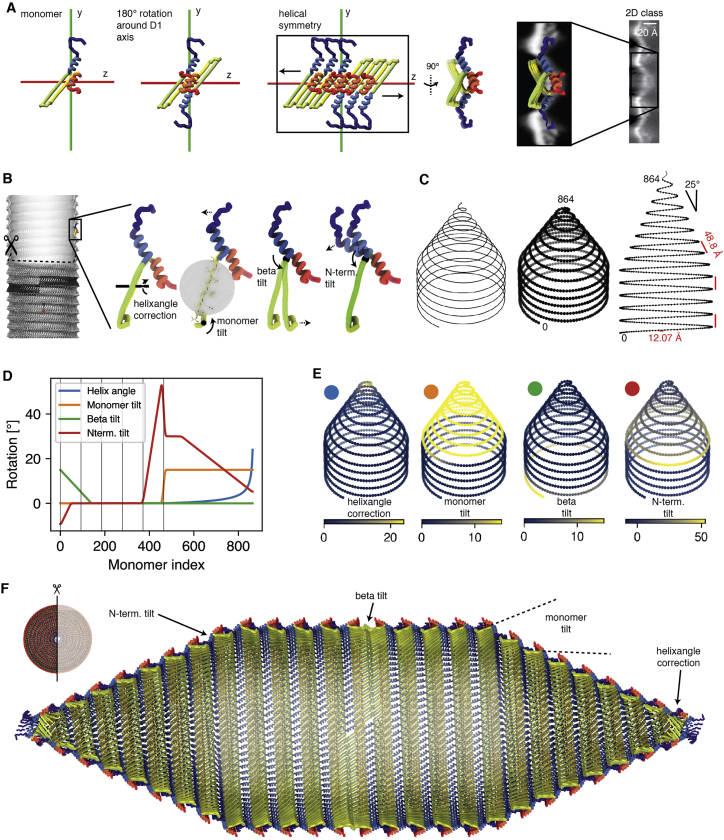
Construction of pseudo-atomic model of a whole *B. megaterium* gas vesicle, related to Figure 3 (A) A GvpA2 monomer was placed next to the symmetry axis (along x) such that a 180° rotation would reproduce a view corresponding to the experimental 2D class average. The β-sheets meet in an angle at this stage, which is later corrected by tilting the sheets. (B) Four rotation parameters (helixangle correction, monomer tilt, β-hairpin tilt and N-term. tilt) used in the model are visualized. (C) The model is based on a helical curve in space with a linearly decreasing radius in the cones. The pitch in both the cylinder and cone is 48.8 Å. 835 monomers are placed equidistantly on the curve with an intermonomer distance of 12.07 Å. (D) The helixangle parameter was extracted from the curve, while the other three parameters were manually tuned to fit the 2D class average data, make the β-sheets line up in the PRP and avoid gaps by adjusting the N-termini. Vertical lines every 93 units depict the monomers on each wrung of the cylindrical part. (E) Parameters mapped onto the monomers (F) Cross-section of the final model highlighting the impact of the four model parameters. The N-terminal tilt is required to maintain the binding site of the N-terminus to the adjacent rib despite the constricting cone radius. The beta tilt is required to make the orientation of the beta-hairpin compatible with the 2D classes at the seam. The monomer tilt is required to make the monomer orientation compatible with the 2D classes of GV tips. The helixangle correction is required to accommodate the increasing helical angle in the constricting cone toward the tips.

Using restraints from our 3D reconstruction and 2D class averages, we constructed a pseudo-atomic model of a GV half shell. Starting at the PRP, we extended the model from the D1 symmetry axis using the known helical symmetry for the cylindrical part of the GV shell and allowed transitioning into the conical tips by linearly decreasing the radius set by the 25° semi-angle of the cone and while refining structural adaption of GvpA monomers at defined hinge-points to match the experimental data (details in STAR Methods and Figure S3). We then duplicated the half shell by rotating around the D1 axis, leading to a complete GV model consisting of 1,730 monomers and a total molecular mass of 12.2 MDa. Simulated density projections from this model closely match the experimental 2D classes (Figures S2D and S2E).

### Molecular mechanism of gas vesicle biogenesis

Our pseudo-atomic model of the GV assembly shows that half shells interact through contacts at the GvpA β-turns around the circumference of the seam, as well as at the PRP where the GvpA2 rib reverses its polarity (Figure 3C). The pattern of side-by-side contacts between β sheets of GvpA2 monomers (d=d=d=d) along a rib is identical everywhere except at the PRP, where β-hairpins align inversely (d=d-p=p). The PRP is therefore a unique point in the GV assembly that may be recognized by the molecular machinery that facilitates GV growth and is likely the point at which new GvpA molecules are inserted during GV growth. Expansion of the GV assembly could occur either by inserting two oppositely oriented monomers, leading to symmetric expansion of both GV half shells, or by stochastic insertion of monomers into either half shell. If GV growth occurs by insertion of dimers, the PRP should always be located exactly at the midpoint of the cylindrical segment. Instead, we observe that the PRP can be located away from the midpoint of the helical GV segment (Figure 1B), consistent with previous observations.^26^ We therefore propose that insertion of new monomers on either side of the PRP occurs stochastically through ratcheting of the two GV half shells by rotation relative to each other, generating a single monomer gap at the PRP (Figure 3D). This would involve breaking the lateral hydrogen bonds between the two monomers around the PRP, along with breaking and re-establishing hydrophobic contacts (Val_35_, Ile_37_) of the β-turns at the seam with no net loss in energy (Figure 3E). In our model, two factors suggest that the PRP represents the weakest point in the assembly. First, the monomer orientation at the PRP leads to steric hindrance by the α2 helices of the two symmetry-related monomers (Figure 3E). Second, the two oppositely oriented monomers at the PRP are connected by only 6 hydrogen bonds between segments of strand β2 around Val_47_ as compared to 11 hydrogen bonds formed between monomers along the rib (Figure 3G). We propose that the energetic disadvantage of this conformation facilitates opening of the seam for addition of new monomers during growth.

**Figure 4:**
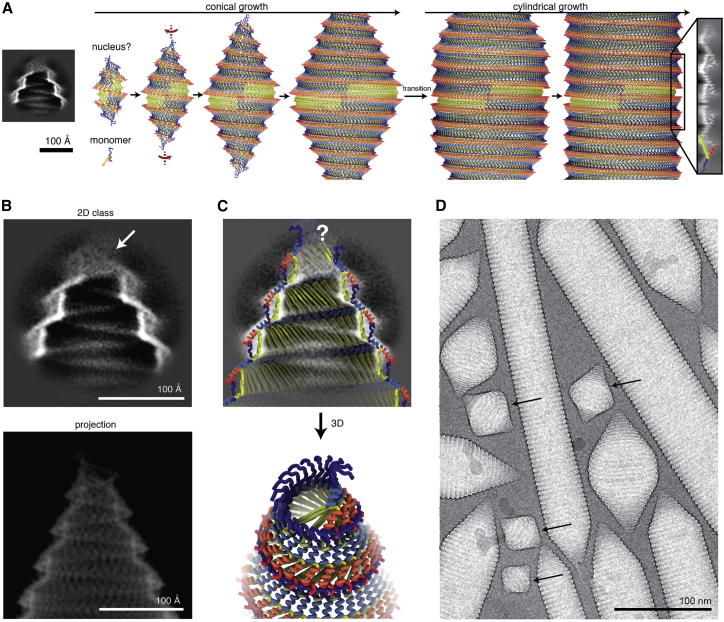
Model of gas vesicle growth from an initial nucleus (A) GV growth from a hypothetical nucleus to mature GVs. The transition from conical to cylindrical growth is annotated. A 2D class of GV tips with proposed nucleus remnants is shown to scale. (B) Class average of the GV tip shows no molecular order at diameters lower than 50 Å toward the cone end (top). 2D projection of the pseudo-atomic model is shown to scale (bottom). (C) 2D class overlaid with cut-through of the pseudo-atomic model (top) and 3D view onto the tip (bottom). The simplified model of a helix with linearly decreasing radius breaks down at the very tip, leading to steric clashes between the main chains. N termini from GvpA monomers at the tip come into proximity and might close off the gas space. (D) Cryo-EM micrograph with immature GV bicones (arrows). See also Video S2.

If the GV grows by adding monomers at the PRP, backward extrapolation starting from a mature GV leads to a state at which the seam forms between two conical half shells (Figure 4A). This is the point where conical growth transitions into cylindrical growth. This transition requires adaption of the relative orientation of adjacent ribs, which we propose is mediated through a hinging motion of the N terminus relative to helix α1 to accommodate for the reduction in diameter at the transition point and along the cone toward the tip (Figure 3H).

Continuing this extrapolation leads to a biconical nucleus that must initially form to start GV biogenesis. According to our model, the original nucleus would remain at the cone tips on both half shells after maturation (Video S2). The 2D classes and the fitted pseudo-atomic model suggest that N-terminal residues of GvpA2 monomers crowd together at the tip (Figures 4B and 4C). At diameters lower than 50 Å, the 2D class averages of these cone tips display weak density (Figure S2F) sealing off the opening. The fuzzy appearance of the density at the tip is indicative of structural heterogeneity in the nucleating monomers.

Video S2. Artist representation of gas vesicle growth, related to Figure 4 The movie illustrates the mechanism of gas vesicle growth by monomer insertion, with the original bicone highlighted in brighter colors. The initial bicones ends form the tips of the mature gas vesicle after conical growth followed by cylindrical growth.

### Conservation of gas vesicle shell architecture

**Figure 5:**
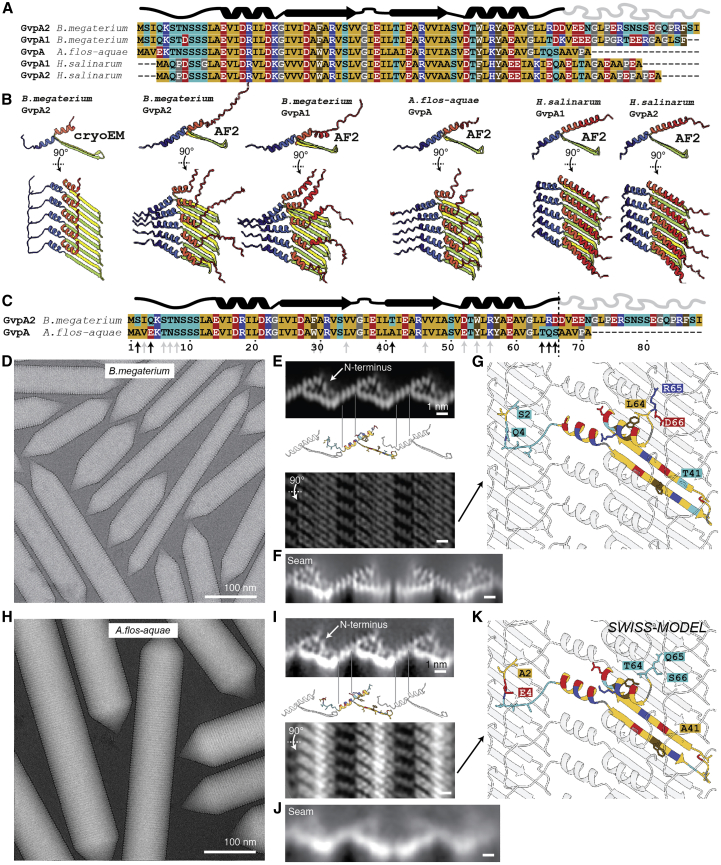
Highly conserved GvpA from *B. megaterium* and *A. flos-aquae* adopt the same fold and assembly (A) Sequence alignment of *B. megaterium* (Mega) GvpA1 (GvpA) and GvpA2 (GvpB), *A. flos-aquae* (Ana) GvpA, and *H. salinarum* (Halo) GvpA1 and GvpA2 show high degree of conservation despite forming GVs of different diameters. (B) AF2 prediction of GvpA 5-mers for selected genes in (A) compared to the cryo-EM structure. Only the middle monomer is shown in side view. AF2 predicts the general arrangement of a GvpA rib and the angle between the β-hairpin and α helix 1 accurately. The conformation of the N-terminal coil appears different, and the distance between α helix 1 (where gas pores are located) is not accurately modeled by AF2. (C) Protein primary sequences of wall-forming protein GvpA from both *B. megaterium* and *A. flos-aquae* are very similar. Black arrows show 6 property-changing mutations; gray arrows show 12 property-conserving mutations in the ordered part of the structure. (D) Representative cryo-EM micrograph of *B. megaterium* GVs. (E) 2D-projected side view and top view of the 3.2 Å cryo-EM density of *B. megaterium* GVs. (F) 2D class average of seam between two GV half shells. (G) Atomic model of *B. megaterium* GV wall with different side chains between both species displayed and property-changing mutations highlighted by one-letter-code. Residues are colored according to side chain chemistry as indicated in (A). (H–K) Equivalent data for *A. flos-aquae* GVs shows the high degree of conservation of GvpA fold and assembly. The 2D views in (I) are computed by 2D classification of GV edges and collapsed GVs and have a resolution better than 5.4 Å or 4.8 Å as the α-helical pitch or the β strands are resolved. The homology model of the *A. flos-aquae* GV wall was computed using SWISS-MODEL.^27^ See also Figure S4.

Sequence alignments between GvpAs of three evolutionary diverse bacterial and archaeal species producing GVs reveal a high degree of sequence conservation in the structural parts of GvpA, suggesting that the overall mode of GvpA shell assembly must be similar. This is supported by computational predictions using AlphaFold2, revealing highly similar assemblies (ribs) of GvpA oligomers for the evolutionarily distant GvpAs in firmicutes (*B. megaterium*), cyanobacteria (*A. flos-aquae*) and the archaeon *H. salinarum*, that all resemble our experimental structure (Figures 5A and 5B).

**Figure S4:**
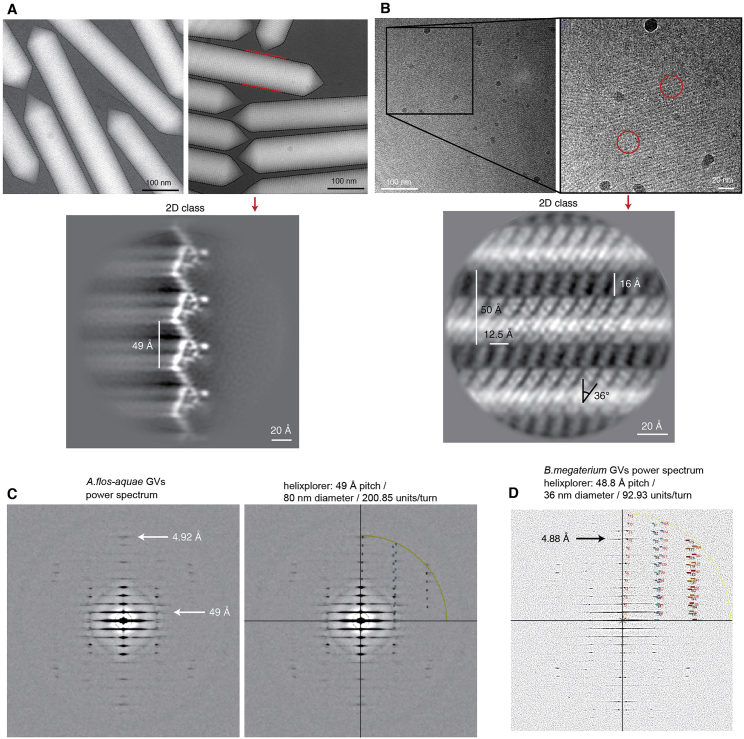
Cryo-EM of *A. flos-aquae* gas vesicles, related to Figure 5 (A) Representative micrographs of *A.flos-aquae* GVs. GV edges were analyzed by 2D class averaging to give a low-noise high-resolution 2D view of the edges, to reveal a repetitive zigzag pattern. The 2D view shows details of at least 5.4 Å resolution corresponding to the α-helical pitch. (B) The same dataset contained collapsed GV wall segments. Those can be averaged as well by 2D class averaging to reveal a high-resolution top-view of the GV wall with better than 4.8 Å resolution as the β-strands are resolved. (C) Computing the sum of in-plane rotated power spectra of segments of all GVs in the dataset gives rise to a layer-line pattern typical for helical assemblies. This approach can be seen as a form of fiber diffraction where fibers are aligned computationally. Overlay with computed layer line patters (Helixplorer, http://rico.ibs.fr/helixplorer/) shows good agreement to a helix with 49 Å pitch and 200.85 units per helical turn. (D) Power spectrum of *B.megaterium* power spectrum with first layer line at 48.8 Å. The upper right quadrant is overlaid by a screenshot from Helixplorer (http://rico.ibs.fr/helixplorer/) used for interactive exploration of helical symmetry.

To verify these predictions, we employed cryo-EM to image GVs of *A. flos-aquae*. Differences in the GvpA sequences between *A. flos-aquae* and *B. megaterium* GvpA2 locate mainly to the N-terminal and C-terminal regions of the folded core (Figure 5C). *A. flos-aquae* GVs formed cone-ended cylinders with mean diameter of 87 ± 7 nm, consistent with previous observations.^18^ Despite significant effort, 3D refinement using a similar helical reconstruction strategy as for *B. megaterium* did not converge. Closer inspection of full-length GVs revealed them to flatten in the thin ice of the cryo-EM sample, breaking symmetry assumptions of helical reconstruction. We therefore resorted to obtain high-resolution structural information from 2D classification. Class averages of GV edges showed a corrugated zigzag pattern of the ∼2 nm-thick wall formed by GvpA (Figure S4A). Another set of 2D class averages, obtained from collapsed GVs also present in the data, revealed the *A. flos-aquae* GV wall to consist of a periodic array of ribs consisting of dense 5.0 × 1.25 nm GvpA subunits tilted at −36° relative to the long axis of the cylinder (Figure S4B). The class averages with a resolution of better than 4.8 Å allow discerning the α-helical repeat of helix α1 bridging the gap of adjacent ribs and the individual β strands of the polymerizing β-hairpin. A cumulative Fourier spectrum computed from in-plane rotated GV segments showed a typical Fourier transform of a helix (Figure S4C), consistent with our 3D reconstruction from *B. megaterium* GVs (Figure S4D). We compared the *A. flos-aquae* 2D classes of GV wall side and top views with equivalent projections of the *B. megaterium* GV structure. On the level of the main-chain fold, the two GV shell assemblies are indistinguishable, hence confirming the conserved architecture of GvpA assembly of the GV shell (Figure 5). Our model of two contacting half shells also applies to *A. flos-aquae* GVs as we observe similar contacting β-turns in 2D class averages of the seam (Figures 5F and 5J).

**Figure S5:**
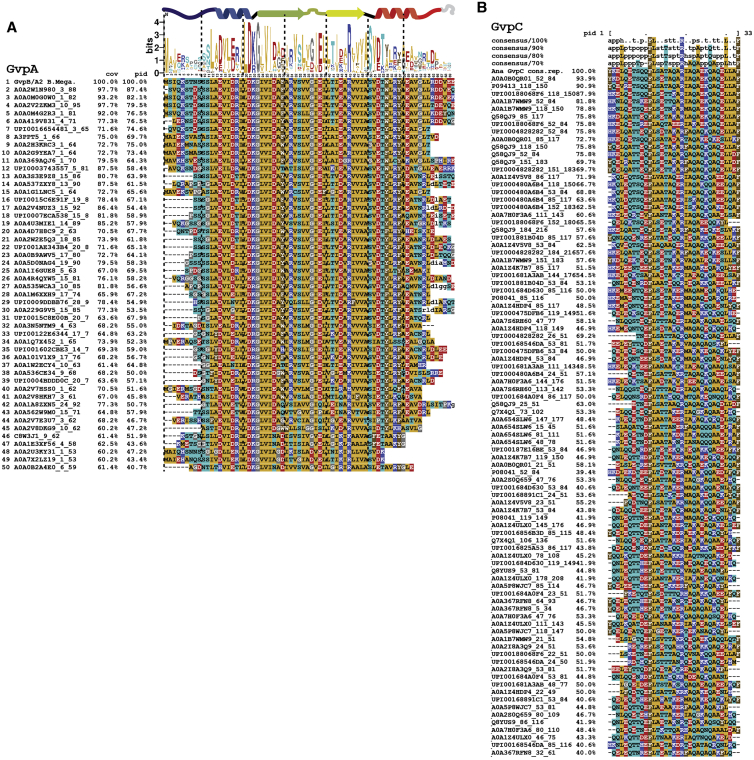
Evolutionary conservation of gas vesicle proteins, related to Figure 6 (A) Multiple sequence alignment of *B.megaterium* GvpA2 with 50 sampled Uniref. 90 clusters of ∼40–100% sequence identity. (B) MSA of sequences similar to *A.flos-aquae* GvpC 33 AA repeats. Alignment of 33 amino acid repeat consensus sequence of Ana GvpC with 91 similar sequences from Uniref. 90 clusters with ∼40–100% sequence identity reveals a highly conserved pattern including leucine, phenylalanine and Arg19 residues. The consensus sequences on top were computed in MView and show residues conserved on at least 70%, 80%, 90% and 100% of sequences.

More generally, our results establish that 2D classification of GV edges in cryo-EM data can give valuable structural insight into GV architecture for cases when 3D reconstruction is out of reach. This is due to the small unit cell of the GV wall, where not many structural features overlap in side views at the GV edges. 2D class averages can be computed to sufficient resolution to see secondary structure elements and even large side chains. We suggest that this approach can be used for comparative studies of GVs from different species with larger sequence divergence to reveal different assembly modes, such as resulting from differences in binding modes of the evolutionarily less conserved GvpA N terminus (Figure S5A).

### Reinforcement of the gas vesicle shell by GvpC

**Figure 6:**
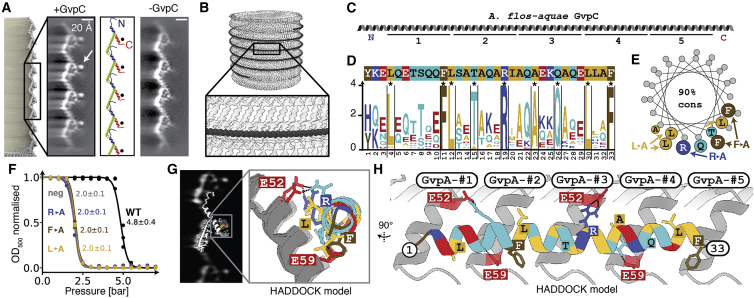
The secondary wall protein GvpC binds along the GvpA ribs of *A. flos-aquae* GVs (A) Comparison of 2D class averages of GV edges with (left) and without (right) GvpC reveals an additional circular density (arrow). A cartoon model of GvpA helps locating the GvpC density to helix α2 (red). (B) Artist impression of GvpC molecules wrapping around GVs. (C) Predicted secondary structure and 33-amino-acid repeats 1–5 of *A. flos-aquae* GvpC. (D) Consensus sequence of the GvpC repeats with logo representation of evolutionary conservation reveals nine highly conserved residues. The height of the characters depicts the degree of conservation (information content in bits). (E) Helical wheel plot of highly conserved (>90% conserved in 91 GvpC sequences from other species) amino acids reveal that there is one highly conserved face of the α-helical repeat. Experimentally tested GvpC mutants are indicated. (F) Critical collapse pressure measurements of *A. flos-aquae* GVs supplemented with wild-type GvpC or GvpC mutants or stripped of GvpC. (G) Comparison of 2D class average of GvpC-bound GVs and predicted binding mode between a GvpC consensus repeat and seven *A. flos-aquae* GvpA monomers. (H) Rotated view of binding model with predicted interactions of residues. See also Figures S5 and S6.

Many GV gene clusters contain a second structural GV protein GvpC, which is absent from our model GV from *B. megaterium*.^28^ GvpC binds on the outside of GVs and increases the critical collapse pressure of GVs.^9^^,^^29^ While the essential role of GvpC has been firmly established, how it binds to and reinforces GVs has remained elusive. We acquired cryo-EM datasets of *A. flos-aquae* GVs, and to investigate the structural role of GvpC, we imaged the GVs in the presence and absence of GvpC and compared 2D class averages of the GV edges (Figure 6A).

**Figure S6:**
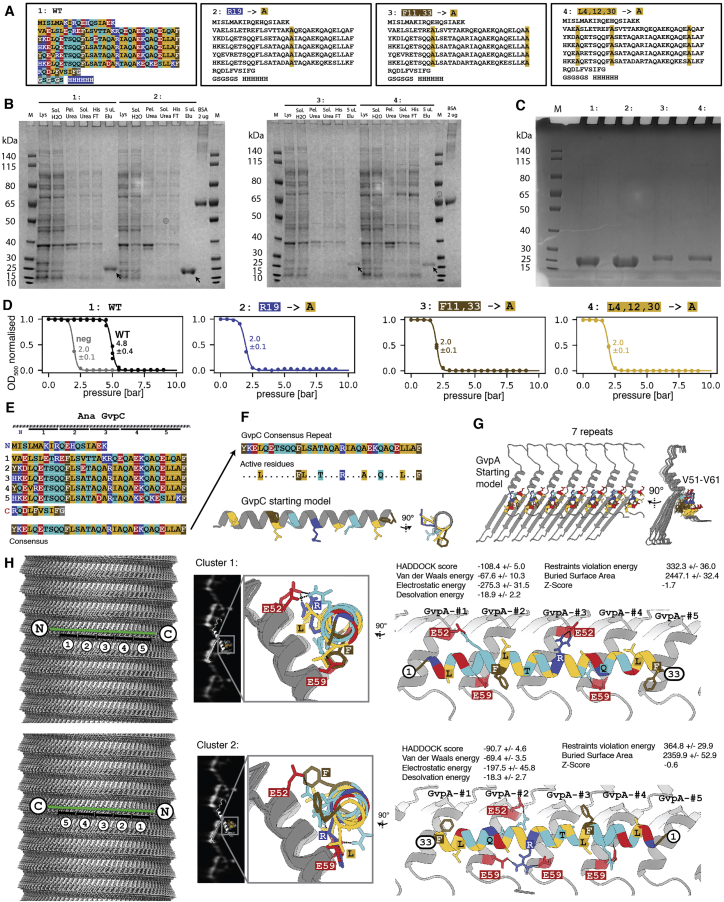
Binding analysis of GvpC to *A.flos-aquae* gas vesicles, related to Figure 6 (A) A GvpC wild-type construct and three mutants were designed with F, L and R residues in all five repeats mutated to alanine. All constructs have a C-terminal GSGSGS linker and 6x His-tag. (B) SDS-PAGE following purification steps of wild-type GvpC and point mutants. Arrow indicates final product. Legend as follows: P:Pellet/SolH2O:Soluble fraction in aqueous solution/Pel.Urea:Pelleting fraction in urea-containing buffer/Sol.Urea:Soluble fraction in urea-containing buffer/HisFT:Flowthrough from Ni-NTA column/5uLElu:Eluted fraction from Ni-NTA column (C) Overloading an SDS-PAGE gel with GvpC mutant sample indicates high degree of purity. (D) Collapse pressure measurements of the four constructs confirm binding to *A.flos-aquae* GVs of the wild-type construct and loss of binding for the mutants. Stripped GVs before re-addition of GvpC were measured as a negative control. Circles are measurement points from three independent re-addition experiments. Solid curves are fits of a sigmoid function, with the stated number being the pressure when the normalized OD500 drops to 0.5. (E) Primary sequence of Ana GvpC with 5 repeats and the consensus sequence of the repeats. (F) Identified highly conserved residues in the repeat are highlighted and chosen as ’active residues’ in HADDOCK. A perfect α-helical peptide is used as the starting model. (G) Amino acids V51 to V61 on α-helix 2 are chosen as the active residues (on a homology model of GvpA for *A.flos-aquae*) based on observed close proximity in 2D class averages. GvpA was repeated 7 times according to the helical symmetry of the solved *B.megaterium* assembly. (H) Two possible binding geometries of GvpC are shown on a *B.megaterium* GV density oriented with the seam on the bottom and the tip on the top: direction from C to N-terminus is following the left-handed loop of the helix, or reverse. HADDOCK protein docking results between GvpC and GvpA fall into two main clusters, one highest-scoring solution with a score of −108.4 and a solution with reverse binding polarity of GvpC with a score of −90.7. The best solution of each cluster is shown. The solution was fitted into 2D class averages of *A.flos-aquae* edges.

The class average containing GvpC shows an additional dot-like density (∼10 Å diameter) located in vicinity to helix α2 (Figure 6A). This feature and its dimensions are consistent with the projection of an α helix viewed along its helical axis. Indeed, GvpC is predicted to be all α-helical^10^ and consists of five 33-residue repeats (Figures 6B and 6C) that are highly similar in sequence. The class averages suggest that GvpC binds along the ribs of GVs and contacts helix α2 of GvpA (Figure 6B), which is supported by a molecular envelope of the *A. flos-aquae* GV wall determined by electron tomography.^30^ We analyzed the evolutionary conservation of the repeat sequence. In a set of 91 GvpC sequences (Figure S5B) from different organisms, we find nine residues to be conserved in more than 90% of those sequences, including a strongly conserved set of leucine (Leu_4,12,30_), phenylalanine (Phe_11,33_), and arginine (Arg_19_) residues (Figure 6D). A helical wheel plot of the repeat shows that all conserved residues cluster on the same face (Figure 6E) that likely forms the binding interface. We further confirmed the importance of these residues by designing alanine mutants for occurrences in all five repeats (Figure S6A). All GvpC mutants abrogated stabilization of GVs (Figure 6F).

Our 2D class averages provide strong spatial restraints on the positioning of GvpC relative to the GvpA ribs, while the conservation pattern and mutants pinpoint residues essential for binding. We used these data as restraints to predict a model for GvpC binding by computational docking (Figure 6G and S6E–S6H).

While our data do not allow us to decisively distinguish whether GvpC follows the left-handed spiral of the rib upwards toward the tips, or downwards, we obtained the highest-scoring docking solution with the downward orientation (Figure 6H). A single GvpC repeat spans four monomers of GvpA. In this GvpA tetrad, glutamate residues Glu_52_ and Glu_59_ in GvpA monomers 1, 2, and 4 form hydrogen bonds with GvpC. In our model, Glu_52_ in monomer 3 binds to the conserved Arg_19_ of GvpC, while Glu_59_ in monomer 4 binds to the conserved Gln_26_. The (Leu_4_, Leu_12_, Leu_30_) triplet inserts between the α2 helices of the GvpA tetrad, while Phe_11_ and Phe_33_ are sandwiched in between α2 in GvpA monomers 2 and 3 and face-on on helix α2 of monomer 4.

## Discussion

GVs represent a remarkable example of biomolecular self-assembly. Our results provide a canonical structural framework for the unique molecular properties of GVs, including their selective permeability to gases,^7^^,^^31^ their mechanical stability,^25^ and their distinctive ability to grow without compromising the integrity of its shell.^3^ Our work establishes an atomic resolution model of the mature GV shell formed exclusively by GvpA. A key question is how GvpA nucleates to form an elementary bicone from which the shell extends during GV growth. Besides GvpA, many GV gene clusters contain genes encoding the proteins GvpJ, GvpM, or GvpS^28^ that exhibit high sequence homology and predicted folds similar to that of GvpA. A dominant structural role of these homologs in mature GVs is unlikely, as none of them were found in intact GVs,^29^ suggesting a putative role as nucleation factors. Additional support for this role comes from the observation that, in some species, GvpJ and GvpM are expressed as part of a separate transcript exclusively during early exponential growth,^8^ whereas a transcript of gvpACNO is expressed at later stages, possibly to enlarge the formed nuclei. How the initial nucleus is structured and by which mechanism other Gvps assist in growth remain open questions.^32^

In most organisms, biconical GVs transition their growth mode when reaching a certain diameter and continue extending cylindrically. This transition occurs over a range of diameters, different for each individual GV. We observed mature cylindrical *B. megaterium* GVs with a diameter of 55.5 ± 7.3 nm corresponding to 145 ± 19 monomers per helical turn and mature *A. flos-aquae* GVs with diameter of 87.1 ± 6.9 nm corresponding to 227 ± 18 monomers per helical turn. The mechanism for the bicone-to-cylinder transition is unknown. The GvpA ribs in the cones are highly curved and may be energetically disfavored. Insertion of new monomers in a bicone results in enlargement of the cone and reduces rib curvature. However, the expanding cone does not provide defined interactions between monomers of adjacent ribs. In contrast, cylindrical GV segments have crystalline order where the N terminus always has identical contacts to the adjacent rib. The interplay of curvature preference and the energetic advantage of crystallinity could favor a cone-to-cyclinder transition at a certain critical diameter. This suggests that the GvpA N terminus could play a role in determining the size distribution of GVs. Consistently, the sequence of the N terminus is most divergent. Further evidence for the decisive role of GvpA sequence in defining the final diameter comes from a hybrid GV gene cluster where *A. flos-aquae* GvpA integrated into the native gene cluster of *B. megaterium*, resulting in GVs with diameters consistent with native *A. flos-aquae* GVs.^33^

Our structure reveals that the gas permeability of the GV wall can be ascribed to a large number of molecular pores formed between α1 helices of the GvpA shell, the size of which is compatible with the collision diameters (2.65–3.64 Å) of typical atmospheric gases dissolved in the cytosol.^23^ Surprisingly, the GV wall has been shown to be permeable to perfluorocyclobutane (C_4_F_8_) with a collision diameter (6.3 Å) exceeding the estimated pore size in our structure. While atmospheric gases appear to diffuse freely through the GV wall,^31^ the diffusion coefficient of C_4_F_8_ is consistent with a very small number (∼11) of such pores.^7^ Based on our pseudo-atomic model, such pores would need to be located at the seam, PRP or at the conical tips. Alternatively, flexibility in the GV shell may modulate size of the regular gas pores to allow passage of C_4_F_8_.

We inferred a binding mode of a single 33-amino-acid repeat of GvpC to four consecutive GvpA monomers assembled in the cylindrical *A. flos-aquae* GV shell. The requirement for a binding site comprised of consecutive GvpA monomers in a helical assembly is consistent with previous observations in split-GFP assays showing that monomeric GvpA and GvpC do not interact.^32^ What remains open is how the five consecutive GvpC repeats are structured. It is likely that all repeats would form identical molecular contacts with the GvpA ribs. The side-by-side distance of GvpA monomers measured between identical points of the inner β-hairpins is 12.1 Å. Due to the curvature of GVs, this distance increases radially outwards across the GV wall toward the binding site for GvpC. For an average 87 nm *A. flos-aquae* GV, this distance is ∼12.7 Å. A single perfectly α-helical repeat of GvpC would span 49.5 Å (33 × 1.5 Å/residue), only slightly shorter than the 50.8 Å spanned by a stretch of four GvpA monomers. The five tandem repeats of native GvpC would bind to 20 GvpA monomers. Taking into account additional space for the N and C termini of GvpC, this corresponds well to a previously determined 1:25 M ratio of GvpA to GvpC.^10^ The modest distance mismatch between one GvpC repeat and a GvpA tetrad could be accommodated by slight deviation of GvpC from perfect helicity. It has been previously suggested that this would also be required to maintain the relative orientation of consecutive repeats, as with perfect helicity they would be (100° × 33) % 360 = 60° rotated toward each other.^10^ A small unstructured stretch would allow GvpC to adapt to different curvatures of the GvpA ribs in cylindrical and conical parts and in GVs of different diameters.

We show that GvpC binds to helix α2 of the GV wall, thereby increasing the critical collapse pressure. The wall thickness measured between the extremes of the corrugation pattern increases from ∼3 nm to ∼4 nm when GvpC is bound. Because the buckling pressure of thin-walled cylinders is proportional to the third power of the wall thickness,^25^ the most straightforward explanation for enhanced stability is that GvpC bound on the outside of GVs increases the wall thickness. The real picture is likely more complicated. Our structure reveals that GVs are assembled from two helical half shells that are connected by a seam. Because of its structure, it is conceivable that the seam is a weak point of the GV where failure will occur first. In this case, GvpC would be most critical to prevent failure at the seam, which questions its requirement elsewhere on the GV shell.

Recently, GVs have been repurposed as genetically encoded acoustic reporters.^34^^,^^35^ The high contrast in density between gas-filled GVs and surrounding cellular structures makes them amenable to ultrasound imaging.^36^ While native GVs display little shell deformation under ultrasound exposure, GVs that are stripped of GvpC become less stiff and scatter non-linearly above a certain pressure threshold.^37^ This behavior enables amplitude modulation imaging and multiplexing of stripped and unstripped GVs in an *in vivo* context.^37^^,^^38^ Engineering conditional binding strength of GvpC can transform GVs into biosensors with switchable acoustic properties.^39^^,^^40^ Our insights into GvpA-GvpC interaction provide a rational basis for designing such sensors. Moreover, the high-resolution structure of the GvpA shell may enable development of designer GVs with custom mechanical shell properties through direct engineering of the GvpA sequence.

Together, our results establish the molecular basis of a widely conserved buoyancy-controlled motility apparatus in aquatic bacteria and archaea. Our study will form the foundation for addressing a multitude of open questions on GV biogenesis such as nucleation, growth, width regulation, function of other GV gene products in GV assembly, and species-to-species variability of GV gene clusters.

### Limitations of the study

While our structure provides an atomic model of the main structural protein GvpA as it forms the GV wall assembly and our data allowed inferring the binding geometry of GvpC to the GV wall, the function and/or structural role of other accessory proteins in GV gene clusters in GV biogenesis is yet to be determined. Some of those proteins presumably work together to create a nucleus whose precise structure remains unknown.

We also report a pseudo-atomic model of an entire GV. The precise structure of the GvpA monomer in this model is supported by cryo-EM data at 3.2 Å resolution; however, the special structural features of the assembly such as the seam, polarity reversal point, and tip are inferred from 2D projection views alone. The exact molecular details at these special positions can slightly deviate from the pseudo-atomic model we present.

## STAR★Methods

### Key resources table

**Table:** 

REAGENT or RESOURCE	SOURCE	IDENTIFIER
**Bacterial and virus strains**		

BL21 (DE3) pLysS	EMBL protein expression core facility	AJLC0003
BL21 (DE3)	EMBL protein expression core facility	AJLC0002
A.flos-aquae	CCAP (Culture Collection of Algae and Protozoa)	CCAP 1403/13F

**Chemicals, peptides, and recombinant proteins**		

SoluLyse	genlantis	L100125
(Isopropyl β-D-1-thiogalactopyranoside) IPTG	VWR	CAS 367-93-1
Lysozyme	Roth	CAS 12650-88-3
DNAseI	ITW reagents	CAS 9003-98-9
BG11	Sigma	73,816

**Deposited data**		

Atomic coordinates of *Bacillus megaterium* GvpA2	This study	PDB: 7r1c
Cryo-EM structure of *Bacillus megaterium* gas vesicles (helically symmetric)	This study	EMDB: 14340
Cryo-EM structure of *Bacillus megaterium* gas vesicles (after local refinement)	This study	EMDB: 14238
Pseudo-atomic model of a complete *Bacillus megaterium* gas vesicle	This study	https://doi.org/10.5281/zenodo.6458345
2D classes of gas vesicle seams from cryo-EM micrographs of *Bacillus megaterium* and *Anabaena flos-aquae* gas vesicles	This study	https://doi.org/10.5281/zenodo.6867443
Alphafold2 models of *Anabaena flos-aquae* and *Halobacterium salinarum* GvpA	This study	https://doi.org/10.5281/zenodo.6867443
Atomic models from molecular docking solutions for *Anabaena flos-aquae* GvpC bound to GvpA	This study	https://doi.org/10.5281/zenodo.6867443
Surface models from gas pore analysis in *Bacillus megaterium* gas vesicle	This study	https://doi.org/10.5281/zenodo.6867443

**Recombinant DNA**		

pET28a-GvpC_His6	Genscript, this study	AJLD0162
pET28a-GvpC_His6 (R19A)	Genscript, this study	AJLD0163
pET28a-GvpC_His6 (F11A,F33A)	Genscript, this study	AJLD0164
pET28a-GvpC_His6 (L4A,L12A,L30A)	Genscript, this study	AJLD0166
pST39-pNL29 (Lakshmanan et al.^20^)	Addgene	#91696

**Software and algorithms**		

MOLE 2.5	Sehnal et al.^22^	https://mole.upol.cz/
SWISS-MODEL	Waterhouse et al.^27^	https://swissmodel.expasy.org/
RELION 3.1	Zivanov et al.^41^	https://github.com/3dem/relion
Gctf 1.06	Zhang^42^	https://www.mrc-lmb.cam.ac.uk/kzhang/
cryoSPARC 3.1 and 3.3	Punjani et al.^21^	https://cryosparc.com/download
ChimeraX 1.4	Goddard et al.^43^	https://www.cgl.ucsf.edu/chimerax/download.html
WinCoot 0.9.8.1	Emsley et al.^44^	https://bernhardcl.github.io/coot/wincoot-download.html
Isolde 1.4	Croll^45^	https://isolde.cimr.cam.ac.uk/download/
Phenix 1.13	Liebschner et al.^46^	https://phenix-online.org/download
TOPAZ v.0.23	Bepler et al.^47^	https://github.com/tbepler/topaz
Chimera 1.13.1	Pettersen et al.^48^	https://www.cgl.ucsf.edu/chimera/download.html
AlphaFold2	Jumper et al.^49^	https://github.com/deepmind/alphafold
ColabFold	Mirdita et al.^50^	https://github.com/sokrypton/ColabFold
EMBOSS	Rice et al.^51^	https://emboss.sourceforge.net/download/
ConSurf web server	Ashkenazy et al.^52^	https://consurf.tau.ac.il/
HMMer v.3.3.2 web server	Finn et al.^53^	https://www.ebi.ac.uk/Tools/hmmer/
MView	Brown et al.^54^	https://desmid.github.io/mview/
WebLogo	Crooks et al.^55^	https://weblogo.berkeley.edu/logo.cgi
HADDOCK 2.4	van Zundert et al.^56^	https://wenmr.science.uu.nl/haddock2.4/

**Other**		

R2/1 300 mesh holey carbon grids	Quantifoil	N/A

### Resource availability

#### Lead contact

Further information and requests for resources and reagents should be directed to and will be fulfilled by the lead contact, Arjen J. Jakobi (a.jakobi@tudelft.nl).

#### Materials availability

Plasmids for GvpC mutants are freely available on request.

### Experimental model and subject details

#### *Escherichia coli* BL21(DE3)pLysS

The *E.coli* BL21 host strain expressing T7 RNA polymerase and T7 lysozyme was used for heterologous production of B.megaterium GVs. *E.coli* BL21(DE3)pLysS cells were grown in lysogeny broth (LB) containing 0.2% (w/v) glucose at 37°C, or at 30°C after induction of heterologous expression by IPTG.

#### *Anabena flos-aquae* CCAP 1403/13F

*A.flos-aquae* (CCAP 1403/13F), also known as *Dolichospermum flos-aquae*, were grown in G625 medium complemented by BG11 medium (Sigma C3061) for approximately 2 weeks until confluence.

### Method details

#### *B.megaterium* gas vesicle expression and purification

The purification protocol for Mega GVs was derived from Lakshmanan et al.^20^ In brief, BL21(DE3)pLysS *E.coli* cells were transformed with the pST39-pNL29 plasmid (a gift from Mikhail Shapiro; Addgene #91696)^20^ and 1 L of LB containing 0.2% (w/v) glucose was inoculated with 10 mL of overnight culture. The culture was grown at 37°C until OD = 0.5 and GV expression was induced with 20 μM IPTG. Following induction, cells were grown at 30°C for 20 h.

The culture was centrifuged at 500 rcf for 2 h in 50 mL Falcon tubes. The floating fraction was collected with a peristaltic pump. This process was repeated once more. The resulting 25 mL of liquid were lysed chemically by adding 5 mL of SoluLyse reagent per 50 mL of liquid, 0.25 mg/mL lysozyme and 10 μg/mL DNaseI, and slowly rotated for 1 h at room temperature.

GVs were purified in three overnight rounds of floatation separation by centrifugation at 800 rcf in 50 mL Falcon tubes. After each centrifugation step, the GV-containing top layer was gently removed with a pipette after which the GVs were resuspended in PBS containing 6M urea (first round), and subsequently in PBS alone. Final concentration was determined as OD_500_ = 3.1 by optical density measurement at 500 nm against a sonicated blank. The sample was dialyzed into imaging buffer (20 mM Tris, 50 mM NaCl, pH = 8) prior to cryo-EM sample preparation.

#### *A.flos-aquae* gas vesicle purification

The purification protocol for GVs from *A.flos-aquae* was derived from Lakshmanan et al.^20^ Briefly, *A.flos-aquae* (CCAP 1403/13F), also known as *D. flos-aquae*, were grown in 250 mL G625 medium complemented by BG11 medium (Sigma C3061) for approximately 2 weeks until confluence. The culture was centrifuged at 350 rcf for 4 h or until a floating layer of cells was observed. Subnatant was removed using a syringe before lysing the cells in 500 mM sorbitol and 10% v/v lysis buffer (SoluLyse) at 4°C for 6–8 h while gently rotating. GVs were purified by three rounds of flotation separation with 4–6 h centrifugation at 350 rcf. After each centrifugation, subnatant was removed by syringe after which GVs were resuspended in PBS at pH 7.4.

#### GvpC mutant purification

Codon-optimized genes for *A.flos-aquae* wild-type and mutant (R19A; F-11,33-A; L-4,12,30-A) GvpC (Uniprot: P09413) including a C-terminal 'GSGSGS' linker and a C-terminal 6xHis-tag in a pET-28a(+) vector were obtained from Genscript (New Jersey, United States). The mutations were engineered into all five repeats.

Proteins were expressed in *E.coli* BL21-DE3 cells grown in 750 mL autoinduction medium^57^ for 3 h at 37°C before the temperature was lowered to 20°C for additional 20 h. The bacteria were harvested by centrifugation and lysed by freeze-thaw cycles in lysis buffer (20 mM Tris, 500 mM NaCl, 0.1% Triton X-, 20 mM imidazole; 5 mL per gram of pellet). Lysozyme (0.15 mg/mL) and DNAseI (10 μg/mL) were added and the lysate rotated for 2 h at room temperature. Isolation of inclusion body and IMAC purification was performed as described previously.^20^ Protein purity was assessed by SDS-PAGE and concentration was determined according to Bradford.

#### Preparation of gas vesicles with recombinant GvpC and collapse pressure measurements

*A.flos-aquae* GVs were stripped of GvpC by resuspending in 6 M urea and 60 mM Tris buffer, using 3 rounds of flotation separation as previously described.^20^ Recombinant GvpC was added to stripped GVs according to the formulation: 2 x OD_500_ × 198 nM x GV volume (L) = nmol GvpC. GvpC will be present in a 2-fold excess under the assumption of a 1:25 M ratio of GvpC/GvpA.^20^ The urea solution was then slowly replaced with PBS at pH 7.4 by 2 rounds of ∼12 h of dialysis over a 7–10 kDA MWCO dialysis membrane. Finally, 3 rounds of floatation separation at 350 rcf removed traces of urea. GVs were diluted to OD_500_ = 0.1–0.4 for collapse pressure measurements. Samples were loaded in a pressure vessel and hydrostatic pressure was increased in increments of 0.5 bar using pressurized nitrogen gas. Samples were allowed to equilibrate for 5 s after pressure changes before absorption was measured using a spectrophotometer (Ocean optics) at 500 nm OD_500_ values were normalized between the minimum and maximum for each measurement. Three independent re-additions per GvpC mutant were performed and measured.

A sigmoid function with *p*_*0*_ as the inflection point and *k* as the width was fitted to the curves using the means and standard deviations of measured triplicates (n = 3) as input for the scipy *'curve_fit'* function.

$${OD}_{500,norm}=\frac{1}{1+e^{k\left(p-p_{0}\right)}}$$

The error of p_0_ was determined using a bootstrapping approach, performing the fit 50 times with a random set of n out of n measurement points with replacement. The parameters and their uncertainty were estimated as their mean and SD over the 50 fits.

#### Cryo-EM of *B.megaterium* gas vesicles

*B.megaterium* GVs at OD_500_ = 3.1 were applied to a freshly glow-discharged Quantifoil R2/1 grid and frozen using a Leica plunger set to 95% humidity, front-side blotting and 20°C with blot times ranging from 5 to 11 s. Micrographs were collected on a Titan Krios (Thermo Fisher Scientific) microscope at the Netherlands Center for Electron Nanoscopy (NeCEN) operated at 300 kV. Dose-fractionated movies were acquired on a Gatan K3 direct electron detector at a pixel size of 1.37 Å with 60 frames over an exposure of 30 e^−^/Å^2^ and a defocus range from −0.25 to −1.25 μm.

#### Cryo-EM of *A.flos-aquae* gas vesicles

Native *A.flos-aquae* GVs (containing GvpC) at OD_500_ = 13 in imaging buffer (20 mM Tris, 50 mM NaCl, 0.5 mM EDTA, pH = 8) were applied to a freshly glow-discharged Quantifoil R2/1 grid and frozen using a Leica plunger set to backside-blotting, 95% humidity and 20°C with 10 s blot time. 1273 cryo-EM micrographs at 1.288 Å pixel size were acquired on a JEOL 3200 microscope with a K2 detector using 62 e^−^ total exposure over 60 frames. *A.flos-aquae* GVs stripped from GvpC (OD_500_ = 1, in PBS buffer) were prepared and imaged in a similar same way, using 17.6 e^−^ over 50 frames, 1.288 Å pixel size, acquiring 58 micrographs.

#### Data processing and structure determination of *B.megaterium* gas vesicles

4351 collected super-resolution movies were 2x binned and motion-corrected in RELION 3.1.^41^ CTF determination was performed using Gctf 1.06.^42^ 709 micrographs containing thin GVs with a diameter of 42 nm or less were identified manually. 1021 tubes were manually picked in RELION by selecting start and end coordinates. 36,295 overlapping segments with 512 pixels were extracted along the cylindrical sections with a step size of 49 Å (2x binned). 2D classification was done in RELION3.1 with the 'ignore CTFs until first peak' option turned on. The resulting 2D class averages were grouped by projecting them along the helical axis and calculating the rim-to-rim distance (Figure S2B) between the two density maxima. A class with 35.6 nm edge-to-edge distance was selected containing 2911 segments.

Analysis of in-plane rotated power spectra of the segments using Helixplorer (http://rico.ibs.fr/helixplorer/) revealed a likely helical symmetry between 90.92 and 95.92 units per helical rung, with ∼49 Å helical pitch. 2D classification was not sufficient to separate all symmetries and the final set of segments originated from several assemblies with different symmetries.

3D classification starting from a featureless cylinder and imposing candidate symmetry parameters was used to further select for segments adhering to a particular symmetry, leading to 1460 segments with symmetry of 92.93 units per turn. 3D refinement of those particles with CTF refinement and Bayesian polishing led to a resolution of 3.6 Å at FSC = 0.143. Convergence of 3D refinements was only achieved when using a *'tau_fudge'* parameter of 5.

A final round of particle polishing was used to create a new particle stack extracted only from frames 1–20 (0–10 e^−^/Å^2^) of the movies. Particles were exported to cryoSPARC 3.1.0^21^ and high-pass filtered to 100 Å. 3D refinement with the helical reconstruction algorithm implemented in cryoSPARC also led to a reconstruction at 3.6 Å. To account for small deviations from helical symmetry, e.g. by flattening of the tube in ice, a mask encompassing ∼3x9 GvpA2 monomers was created in ChimeraX.^43^ The particle stack expanded by helical symmetry was subjected to focused refinement in cryoSPARC using the mask, which increased the final resolution to 3.2 Å at FSC = 0.143. The final maps were cropped from a box size of 512^3^ voxels to a box size of 128^3^ voxels centered on the refined region. Local resolution was calculated in cryoSPARC 3.1.0^21^ and mapped onto the monomer structure in ChimeraX.^43^

#### Atomic model building and refinement

To build an atomic model of a GvpA2 monomer the final map density was traced *de novo* using COOT.^44^ The monomer was expanded using helical symmetry (rise: 0.525 Å, twist: −3.87°) into a 15 subunit segment (three ribs with 5 monomers each) to account for connections between monomers and manually adjusted in ISOLDE^45^ before automatic real-space refinement in PHENIX 1.13.^46^ We used phenix_real_space_refine with automatic restraint weighting to refine coordinates and atomic displacement factors against the globally sharpened experimental density map using secondary structure and non-crystallographic symmetry restraints. The central monomer of the 15 subunit segment was used as the asymmetric unit for PDB deposition. Renderings of the cryo-EM density and atomic models were made in ChimeraX 1.4.^43^

#### 2D classification of edges, seams and tips

From the *B.megaterium* GVs cryo-EM dataset, several hundred particles of either seams or tips were picked manually and used to generate a template for automated picking in cryoSPARC 3.3.^21^ Particles were high-pass filtered to 100 Å to eliminate the large negative contrast of the gas space in the GV interior. The picked particles were cleaned up by several rounds of 2D classification to give a clean set of particles of either the seam, the PRP or the tips. These particle sets were used to train a neural network for particle picking (TOPAZ v0.23^47^), which was then applied to the micrographs to pick seams, PRPs, and tips. Those particles were cleaned by several rounds of 2D classification and led to the final presented 2D classes. For display, the 2D classes were treated in ImageJ using an 'unsharp mask' filter.

The two cryo-EM datasets of *A.flos-aquae* GVs with and without GvpC were used to obtain 2D class images of the edges leading to side-views of the wall. Movies were imported into cryoSPARC v3.3,^21^ motion-corrected and CTF-estimated. For both dataset, frames were used only until an exposure of ∼15 e^−^/Å^2^ because shrinking of GVs was observed for high exposures leading to GV edges blurring out. A few hundred edges were manually selected for 2D classification to generate picking references for the cryoSPARC filament tracer. Particles were extracted with 192 pixel box size and high-pass filtered to 100 Å. Several rounds of 2D classification and selection of sharp classes led to the final 2D classes of the GV edges with and without GvpC. For display, the 2D classes were treated in ImageJ using an 'unsharp mask' filter.

Similarly, 2D classes of the GvpA lattice from collapsed GVs were calculated from the *A.flos-aquae* GV dataset above including GvpC. For this, the dataset was preprocessed in RELION 3.1,^41^ points on the lattice manually picked to create a 2D class, which was then used for automated particle picking. Particles were extracted with a box size of 128 pixels and aligned with 2D classification to generate a view onto the collapsed GV wall. The biggest class containing ∼34,000 particles was selected and was treated in ImageJ using an 'unsharp mask' filter.

#### Pore analysis

Gas pores in the GV wall were analyzed using MOLE 2.5.^22^ The gap between α1 helices has a slit-type shape, enabling multiple possible routes for gas diffusion. Several start and endpoints of tunnels around both sides of the slit were selected and tunnels were computed with MOLE. The slit was modeled by three tunnels and displayed in Chimera.^48^ The minimal constriction of the tunnel was calculated as the diameter of the smallest sphere in the respective tunnel model.

#### Pseudo-atomic modeling of a complete gas vesicle

The model was generated from the solved atomic model of *B.megaterium* GvpA2. The GvpA2 monomer was placed next to the x axis (D1 axis) manually such that a 180°, symmetry copy operation around the x axis would reproduce a side view of the GV edge compatible with the determined 2D class average.

A left-handed parametric helix in 3D space was defined with the parameter *t* corresponding to turns of the helix:

$$t_{cap}=\frac{r_{\max}}{P\cdot \sin\left(\alpha \right)}$$

$$r\left(t\right)=\left\{ \begin{array}{c} r_{\max},if\;t<t_{cyl}\\ r_{\max}\cdot \left(1-\frac{t-t_{cyl}}{t_{cap}}\right),if\;t\geq t_{cyl} \end{array}\right.$$

x(t)=r(t)·cos⁡(2πt)

y(t)=−r(t)·sin⁡(2πt)

$$z\left(t\right)=\left\{ \begin{array}{c} P\cdot t,if\;t<t_{cyl}\\ P\cdot t_{cyl}+P\cdot \cos\left(\alpha \right)\cdot \left(t-t_{cyl}\right),if\;t\geq t_{cyl} \end{array}\right.$$

where *t*_*caps*_ is the number of turns in the cap, t_cyl_ is a user-parameter of how many cylindrical turns the model should have (5), *P* is the pitch of the helix of 48.8 Å, r_max_ the radius of the assembly of 178.4 Å, and *α* is the cone angle of the tip (25°).

The starting point of the curve was adjusted to go through the pivot point (in the center of the two β-sheets between amino acid (AA) 28 and AA 42 carbonyl oxygen atom) of the placed monomer by applying a shift along the z axis and rotation around the z axis. Points were placed along the curve at a distance of 12.07 Å, calculated as √[(2πr)^2^ + P^2^]/ut, where ut is the number of monomers per turn defined by the solved helical symmetry (92.93). 835 points were placed with the last 4 points toward the tip being omitted.

A model only with placement of monomers does not reflect the experimental 2D classes well. Four additional parameters were introduced rotating and modifying the monomer.

(1) a correction for the change of helix angle toward the ends of the tip as the helix becomes steeper with narrower radius. The rotation center is between the AA28 and AA42 carbonyl oxygen atoms, and the rotation axis normal to a fitted plane through C-α atoms of all amino acids of the β-hairpin (AA23-49).
(2) a rotation of the entire monomer to account for the tilt of monomers following the cone angle. The rotation center is carbonyl oxygen 36 and the axis normal to the plane of AA24-33 C-β atoms.
(3) a hinging motion of the two β-strands to account for the deformation visible in 2D classes of the PRP where GvpA ribs from both GVs halves meet. The rotation axis is defined by a line between the AA23 C-α atom and the AA49 C-α atom, and moves the atoms of AA23-49.
(4) a hinging motion of the α-helix 1 and the N-terminus, to account for gaps in the assembly formed when monomers tilt toward the cone angle. The axis is defined as in (3), and the rotation moves the atoms of AA2-23.

For the purpose of illustrating GV growth from nuclei to mature cylinders, the model was replicated in Blender 3.0 in a simplified form (without modifications of the monomer) and animated using varying parameters for the diameter and the t_cyl_ parameter.

#### AlphaFold2 structure predictions

Complexes of five monomers of GvpA corresponding to a rib section of the GV wall were predicted using AlphaFold2^49^ run in a Google Colab environment^50^ with each five generated models per run and six recycles. The highest-scoring model was selected and displayed in ChimeraX.^43^

#### Bioinformatics analysis

The sequences of the five 33 AA repeats of *A.flos-aquae* GvpC (Uniprot P09413) was converted into a consensus sequence 'YKELQETSQQFLSATAQARIAQAEKQAQELLAF' using the software 'cons' from the EMBOSS package.^51^ The sequence was used as input for the ConSurf Server^52^ to search the Uniref. 90 database with the HMMER web server^53^ using one iteration, resulting in 91 sequences. The resulting sequence alignment was displayed and consensus sequences calculated in MView.^54^ A sequence logo was calculated from the sequence alignment using WebLogo.^55^ Helical wheel plots were generated in Heliquest.^58^

#### HADDOCK modeling of GvpC binding to *A.flos-aquae* gas vesicles

A α-helical model of the 33 residue consensus repeat of *A.flos-aquae* GvpC ('YKELQETSQQFLSATAQARIAQAEKQAQELLAF') was generated in ChimeraX^43^ using ideal α-helical backbone dihedral angles φ = −57° and ψ = −47°. A homology model of the *A.flos-aquae* GvpA monomer was generated using SWISS-MODEL^27^ based on the structure of *B.megaterium* structure GvpA2 and the *A.flos-aquae* sequence for GvpA (UniProt: P10397). The homology model was extended with the symmetry parameters from the *B.megaterium* assembly in ChimeraX^43^ into a rib of 7 adjacent monomers.

Both models were used as input for HADDOCK 2.4.^56^ For GvpC, residues 4,11,12,15,19,23,26,30,33, which all are >90% conserved in the bioinformatics analysis were chosen as active residues. For GvpA, residues 51–61 of GvpA, part of helix α2 and adjacent to the GvpC density in the 2D classes, were chosen as active residues. All remaining settings were used at default values.

The highest scoring cluster of the docking solutions (HADDOCK score: −108.4 ± 5.0) showed GvpC binding across several GvpA monomers along the helical spiral of the rib, with the GvpC sequence oriented inversely to the direction of helical propagation (with the direction from seam to tip). A very similar cluster was found shifted by one GvpA monomer laterally with the same molecular contacts and was discarded. A second, lower scoring type of cluster (HADDOCK score: −90.7 ± 4.6) showed GvpC binding mode with the GvpC sequence oriented to the direction of helical propagation.

Docking clusters were displayed in ChimeraX^43^ and hydrogen bonds between GvpA and GvpC highlighted with the 'hbonds' command. The highest-scoring solution was manually fitted into a 2D class of the *A. flos-aquae* GV wall with GvpC.

### Quantification and statistical analysis

#### Gas vesicle width measurements

Diameters of *A.flos-aquae* and *B.megaterium* GVs were determined from measurements of the widths of GV projection images from 20 cryo-EM micrographs each for both species. Figure 1E displays all individual measurement data points. Stated are mean and SD of the distributions.

#### Collapse pressure measurements

The collapse pressure of *A.flos-aquae* GVs supplemented with different GvpC mutants (Figure 6F and S6D) was determined as described in the STAR Methods details (section “Preparation of GVs with recombinant GvpC and collapse pressure measurements”). Displayed in Figure 6F and S6D are the collapse pressures and their standard deviations determined in a bootstrapping approach.

## Data Availability

• The refined atomic model of *B.megaterium* GvpA2 (GvpB) has been deposited in the PDB under accession code PDB: 7R1C. The cryo-EM density of a subsection of the *B.megaterium* wall (after local refinement) is available in the Electron Microscopy DataBank under accession code EMDB: EMD-14238. A symmetrized density of an entire GV cylinder is available under accession code EMD-14340. The presented pseudo-atomic model of a complete *B.megaterium* GV is available on Zenodo (https://doi.org/10.5281/zenodo.6458345). 2D class averages of *A.flos-aquae* and *B.megaterium* GVs, predicted gas pores, AlphaFold2 structure predictions and computational docking results of *A.flos-aquae* GvpA with GvpC are also available on Zenodo (https://doi.org/10.5281/zenodo.6867443). • This paper does not report original code. • Any additional information required to reanalyze the data reported in this paper is available from the lead contact upon request.

## Supplemental information

Table S1. Cryo-EM data collection and model refinement statistics, related to Figure 2
