# Supplementary material for: Cryo-EM structure of gas vesicles for buoyancy-controlled motility
Source: Cell. 2023 Mar 2;186(5):975–986.e13. doi: 10.1016/j.cell.2023.01.041 (PMC9994262; doi:10.1016/j.cell.2023.01.041)
Supplement: Table S1. Cryo-EM data collection and model refinement statistics, related to Figure 2 [file mmc3.pdf]

Supplementary Table S1: Cryo-EM data collection and model refinement statistics. Related to Figure 2.

**Data collection**	
Microscope	Titan Krios (Thermo Fisher Scientific)
Magnification	64,000
Voltage (kV)	300
Electron exposure ($e^-/Å^2$)	30
Exposure time (s)	2.4
Number of fractions	60
Number of movies	4351
Defocus range (µm)	0.25-1.25
Pixel size (Å)	1.37
Detector	K3 (Gatan)
Dose rate detector (e^-/pix/s)	24
**Data processing**	
Helical rise (Å)	0.525
Helical twist (°)	-3.874
Final no. of asym. Units	135,780
Global map resolution (Å) (FSC=0.5, unmasked/masked)	4.2/3.6
Global map resolution (Å) (FSC=0.143, unmasked/masked)	3.6/3.2
Resolution range (local, Å)	3.0-3.5
Map sharpening B-factor ($Å^2$)	60.8
Map sharpening method	Global
**Model refinement**	
Model starting point	De-novo
Sequence	GvpB (GvpA2) – Uniprot O68677
Model composition	
Non-hydrogen atoms	497
Protein residues	65
Validation	
MolProbity score	0.69
Clashscore	0
Rotamer outliers (%)	0
C-beta deviations	1
Bond angles RMSD (°)	0.87
Bond lengths RMSD (Å)	0.52
B-factors ($Å^2$)	77-107
CC values (CC mask/CC volume)	0.76/0.75
Ramachandran plot	
Favored (residues)	61
Allowed (residues)	1
Disallowed (residues)	1 (V35)
